# Patellofemoral Pain Syndrome in Young Female Athletes: A Case-Control Study

**DOI:** 10.1155/2022/1907975

**Published:** 2022-04-15

**Authors:** Vito Pavone, Andrea Vescio, Flora Maria Chiara Panvini, Ludovico Lucenti, Alessia Caldaci, Marco Sapienza, Federico Canavese, Gianluca Testa

**Affiliations:** ^1^Department of General Surgery and Medical Surgical Specialties, Section of Orthopaedics and Traumatology, University Hospital Policlinico-San Marco, University of Catania, Catania, Italy; ^2^Department of Pediatric Orthopedic Surgery, Jeanne de Flandre Hospital, Lille University Centre, Lille, France

## Abstract

**Background:**

Patellofemoral pain syndrome (PFPS) is the most common cause of anterior knee pain in children and adolescents, and it is characterized by highly limiting, recurrent, frontal pain.

**Objectives:**

The purpose of the study is to assess the incidence and onset of PFPS in the young female athletes and to compare it to healthy individuals.

**Methods:**

Between 2017 and 2019, 51 subjects were reviewed and divided in three groups: rhythmic gymnastics athletes (RG; 21 individuals, mean age: 13.8 ± 3.6 years), basketball athletes (BG; 17 individuals, mean age: 14.2 ± 3.1 years), and control group (CG; 13 individuals, mean age: 14.5 ± 4.3 years). All patients underwent physical examination including patellar glide, tilt, grind and apprehension tests, tiptoe and jack tests, Coleman block, and navicular drop tests. The clinical and functional outcomes of the subjects were assessed using the Kujala patellofemoral score (KPS).

**Results:**

In RG patients were recorded 66.7% of normal footprint (NF), 9.5% of cavus feet (FCF), and 23.8% of flatfeet (FFF); 14.8% patellar positive tests, KS = 98.6 ± 13.7. BG patients had 70.6% of NF, 11.8% FCF, and 17.6% of FFF; 23.5% patellar positive tests, KS = 98.3 ± 12.4. CG patients had 61.5% of NF, 7.7% of FCF and 30.8% of FFF; 15.4% patellar positive tests, KPS = 98,9 ± 15.3. No statistically significance was found between the three cohorts of patients.

**Conclusions:**

PFPS is a common pathology; muscular imbalance and overuse could exacerbate pain and discomfort in young female athletes. Our findings show high type and level of sport activity are not related to increase frequency of clinical symptoms related to PFPS.

## 1. Introduction

The patellofemoral pain syndrome (PFPS) represents one of most common causes of anterior knee pain in adolescents and young adults, and it is characterized by peripatellar and retropatellar pain, progressive and exacerbated by specific positions and/or activities [1]. The aetiology of PFPS is unknown, although muscle imbalance, tendonitis, or insertional tendinosis of the extensor apparatus, patellar instability, and chondral and osteochondral damage can be involved in the pathogenesis of the disease. Diagnosis can be challenging, and it has historically been based on detailed subjective and objective assessments, with pain on several special tests, including the patellofemoral compression test, palpation of the patella, and extension of resisted knee [2].

Between 19% and 31% of adolescents complain of anterior knee pain [3] and about 7% suffers from PFPS [4], although these figures may be underestimated because the reported annual incidence of PFPS is between 3% and 40% among adolescents [5, 6].

Sport activity is crucial for children's development, is essential for the social inclusion and psychological well-being of the child, and may prevent future pathologies [7–9]. Moreover, it has been shown that young sport practitioners have improved quality of life, brain cortical excitability, long-term neural adaptation mechanisms, and visuospatial abilities [10–14]. Despite those findings, current data show that sport specialization [2] in female adolescents is associated with increased risk of PFPS when compared to multisport athletes [15]. Moreover, Smith et al. [16], in their metanalysis, have emphasised flatfoot as a biomechanical variable that could act as an additional risk factor for PFPS.

The main objective of this study is to evaluate the incidence of PFPS in young female athletes performing intense sport activities such as rhythmic gymnastics and basketball and to compare it to the general population. The secondary aim of the study is to evaluate whether the clinical symptoms in young female athletes can be influenced by foot disorders or not. Our primary hypothesis is that high impact sport activities have lower functional outcome and more evident clinical presentation compared to general population.

## 2. Materials and Methods

### 2.1. Demographics

Between November 2017 and September 2019, 79 young female athletes were clinically evaluated at the Orthopaedics and Traumatology Unit, A.O.U. Policlinico, San Marco, University of Catania, Catania, Italy.

### 2.2. Inclusion/Exclusion Criteria

Inclusion criteria were as follows: female gender, age between 9 and 17 years old, at least 4 times per week of sport practice (basketball or rhythmic gymnastics), and PFPS or anterior knee pain for at least 3 months.

Exclusion criteria were as follows: overweight and obese females (World Health Organization: body mass index (BMI) for age >2 standard deviation); previous knee injury; previous diagnosis of PFPS; concomitant diagnosis of peripatellar tendonitis or bursitis, plica syndrome, Sinding-Larsen's disease, Osgood–Schlatter's disease, and neuromas; patients diagnosed with neurological or neuromuscular disorder, skeletal dysplasia, congenital or posttraumatic deformity, diabetes, or immunological disease; and skeletal abnormalities.

According to the inclusion and exclusion criteria, 38 subjects were selected and included in the study. According to the main sport activity, rhythmic gymnastics or basketball, the sample was divided in two groups: rhythm group (RG: 21 females (55.3%); mean age: 13.8 ± 3.6 years, range 9–17) and basket group (BG: 17 females (44.7%); mean age: 14.2 ± 3.1 years, range 10–17).

Thirteen individuals with similar baseline characteristics but the presence of previous diagnosis of PFPS were included in the study as the control group (CG: 13 females; mean age: 13.5 ± 4.3 years, range 9–17). The remaining 28 patients (35.4%) were excluded from the analysis due to refusal to be included in the study (*n* = 8; 28.53%), lost to follow-up (*n* = 14; 50%), and presence of concomitant pathology (*n* = 6; 21.42%).

Demographic and clinical data were recorded as follows: sex, underlying pathology, age, BMI, weight Z-scores, sport activity, and surgery. RG, BG, and CG did not differ significantly in their demographics (*P* > 0.05) (Table 1).

### 2.3. Primary Outcome Assessment

The primary outcome assessment composed of three parts, including physical examination, clinical tests, and clinical score. Physical examination, clinical tests, and clinical score were performed by the same group of caregivers (orthopaedic surgeons).

### 2.4. Physical Examination

All patients underwent physical examination. Initially, limb and hindfoot alignment in anteroposterior, lateral, and posteroanterior profile was evaluated. The foot examination was based on the measurement of surface landmarks or bony prominences to depict the location and position of different structures within the foot, including the medial longitudinal arch (MLA). The plantar imprints were assessed using a podoscope.

### 2.5. Clinical Tests

The patellar glide test (PGt) with the knee flexed at 30° and the quadriceps relaxed was performed to evaluate lateral retinacular tightness and the patellar tilt test (PTt), carried out with the patient supine with the knee in full extension, and could also detect a tight lateral retinaculum [17, 18]. The same position was used to perform the patellar grind test and the patellar apprehension test in order to rule out elicits anterior knee pain originated in the patellofemoral articular surfaces [17, 18]. The tip toe test [19] and Jack test [12] were used to rule out rigid flatfoot, while the Coleman block test and the navicular drop test [20] were used to rule out neurological cavus feet.

### 2.6. Clinical Score

Clinical and functional outcomes were assessed using the Kujala patellofemoral score (KPS). The KPS is a patient' reported assessment of patellofemoral disorders that assesses subjective symptoms and functional limitations. It is a scoring questionnaire based on three main criteria: the patient should specifically assess anterior knee pain symptoms, the patient should complete the questionnaire independently to exclude investigator bias and to also make possible the use of the questionnaire in association with outpatient clinics, and the total scores should be easily and quickly calculated. Overall, the questionnaire consists of 13 questions representing the main activities performed daily by most patients and potentially responsible for patellofemoral symptoms. Every question has different options where the scale gives a maximum of 5–10 points each item. The maximum score is 100, and it can vary between 0 (serious limitations in all examined movements) and 100 (absence of symptoms) [21]. We used the KPS adapted and validated in the Italian version [22].

### 2.7. Statistical Analysis

Continuous data are presented as means and standard deviations, as appropriate. The analysis of variance test and Tukey–Kramer method were used to compare the clinical assessment between the three groups. Finally, chi-squared tests were used to verify the homogeneity, the differences of the limb and hindfoot alignment, and answer to the specific tests between the cohorts. The selected threshold for statistical significance was *P* < 0.05. Power calculations of sample size were performed on both the patient and control groups for an alpha value of 0.05. Recommended sample size was 45 and power test was 0.91. All statistical analyses were performed using the 2016 GraphPad Software (GraphPad Inc, San Diego, California).

## 3. Results

### 3.1. Physical Examination

**Figure 1:**
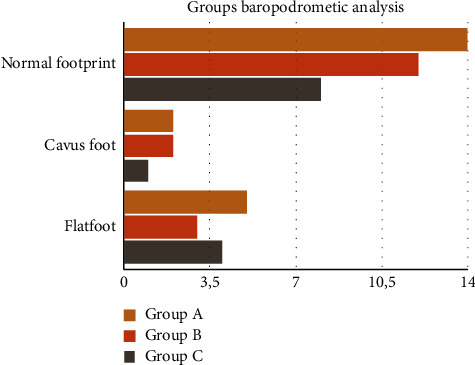
Comparison between groups' baropodometric analysis: flat foot, cavus foot, and normal footprint.

RG (total 21) patients' footprints were normal in 66.7% (*n* = 14) of cases, while 23.8% (*n* = 5) of footprints were compatible with flatfoot and 9.5% (*n* = 2) with cavus foot. BG patients (*n* = 17) had 70.6% (*n* = 12) of normal footprints, 17.6% (*n* = 3) of footprints compatible with flatfoot, and 11.8% (*n* = 2) with cavus foot. CG (*n* = 13) patients had 61.5% (*n* = 8) of normal footprint, 30.8% (*n* = 4) compatible with flatfoot, and 7.7% (*n* = 1) with cavus foot (Figure 1). No statistical significance was found (*P* > 0.99) between the three cohorts of patients (Table 2).

### 3.2. Clinical Tests

**Figure 2:**
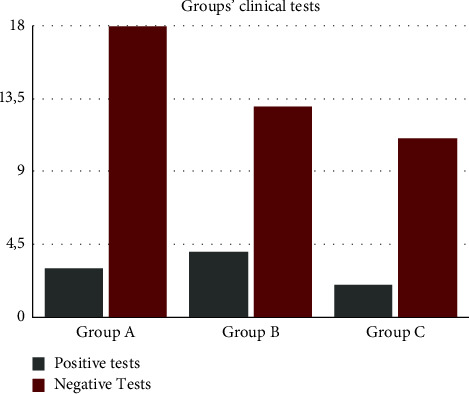
Comparison between positive and negative knee tests in the same groups.

Regarding the clinical tests, 21.6% of the sample showed at least one positive specific anterior knee test, although no differences were found among the three groups of patients (*P* > 0.05). RG patients reported 3 (14.8%) positive tests (2 patellar tilt tests and 1 patellar apprehension tests), while BG patients had 4 (23.5%) positive tests (2 patellar tilt tests and 2 patellar grind tests) (Figure 2). 2 (15.4%) positive patellar tilt tests were recorded in CG patients. No statistical significance was found (*P* > 0.99) between the three cohorts of patients (Table 3).

### 3.3. Clinical Score

According to the KPS system, RG patients had a mean score of 98.6 ± 13.7 (range, 92–100), the BG patients recorded a mean score of 98.3 ± 12.4 (range, 94–100), while CG patients had a mean score of 98.9 ± 15.3 (range, 93–100). No statistically significant difference was found (F_2.55_ = 192.3; *p* = 0.99) between the three cohorts of patients.

## 4. Discussion

This report found that the incidence of PFPS and functional outcome in young female athletes performing intense sport activities is similar to the general population; the sport practice does not trigger PFPS. Despite different muscular training and requests, basketball, or rhythmic gymnastics, players had similar rate of PFPS. Moreover, limb and hindfoot alignment seems to be not particularly involved in the symptomatology in the athletes.

Several studies have reported that patellar maltracking plays a key role in the onset of PFPS [23]; in particular, Keller et al. [24] described PFPS as an “abnormal patellofemoral tracking.” Overall, PFPS is more commonly found in female than male athletes, due to an increased *Q* angle, causing the lateralization of patellar tracking. Although there are studies that show a risk injury sport-related, we found the incidence of PFPS in young female basketball players and gymnasts is comparable to the general population, and type of physical activity is not a trigger for PFPS. Foss et al. [25], for instance, in a study with more than 800 adolescent basketball players, reported above 26% of female players were affected by PFPS, with an overall incidence of 25%.

Current findings show that sport specialization in female adolescents is associated with increased risk of PFPS, when compared to multisport athlete [15, 16]; furthermore, girls and women are at greater risk of knee injuries than boys and men, especially in cutting and jumping sports [26]. Whereby, our analysis focused on two groups of female athletes, basketball players and gymnasts, in competitive teams, compared to a control group. Despite different muscular training and requests, basketball, or rhythmic gymnastics, athletes had similar incidence of PFPS. Moreover, limb and hindfoot alignment seems to be not particularly involved in the onset of symptoms in any of the groups (RG, BG, and CG patients).

The incidence of PFPS in general female population is between 12% and 13% as portrayed in our results (control among 15.4%; sample among 21.5%).

According to a clinical review of the Iranian Female Athletes who wanted to participate in 3^rd^ Iranian Sports Olympiad [27]; the highest PFPS incidence was recorded in rock climbers and volley players (26.3 and 20.4%, respectively). Despite a recent meta-analysis [16] highlighting more than 30% of multiday amateur cyclists (35%) and female ballet dancers (29.3%) complained of symptoms included in PFPS every year, limited data are available for female basketball players and gymnasts.

Although any significant difference could be found between RG and BG patients (14.3% and 23%, respectively), the BG had slightly higher incidence indicating that basketball may play a role in overuse injuries in patients with PFPS [28]. Repetitive running and jumping activities, increase in training, fall on the front of the knee, muscle weakness around the knee and higher up, increase the risk of PFPS in basketball players [16, 27]. To follow a specific training program, change exercise routine and pay attention to prior injuries that might lead to change in the athlete running or jumping, which is of paramount importance.

Gymnasts followed an accurate program of workout where all the muscles, particularly of the thigh, general quadriceps (GQ) and the vastus medialis obliquus (VMO), are intensely trained [29]. Ensuring adequate flexibility of quadriceps and hamstring muscles, as well as building strength of VMO, may reduce the incidence of knee pain [30,31].

Low incidence of PFPS in gymnasts could be related to the training modality, characterized by a high flexibility training program, compared to other high impact sports. However, Daly et al. [29] explained that adolescence could be associated with a higher incidence of injury than adulthood, probably due to the growth process, inducing an imbalance between strength and flexibility.

According to our results, no statistically significant difference was found between RG, BG, and CG patients. In accordance to the KPS system, similar functional outcome was reported in athletes and the general population. Ferreira et al. [32] compared the elite sportsmen and highly active no athletes, reporting minimal differences in knee pain, and hypothesized that outcome could be influenced by the timing of sport season, as previously reported by Herbst et al. [33].

Our study evaluated a group of young female athletes at the beginning of sport season, and it may explain why results were not significantly different.

PFPS is often cited as an overuse injury [34]; the intense period of abnormal or increasing activity and acute fatigue are thought to be risk injury factors [35].

Knicker et al. [36] defined fatigue as an “exercise induced decline of performance;” furthermore, fatigue is known to influence the athlete's optimal performance and to modify the movement patterns [37]. In gymnasts, the control of knee and hip flexion required by some exercises such as landing reduces the forces across the lower extremities; thereby, the incorrect execution of such exercises can increase the risk of injury [38]. In this context, it is fundamental to use prevention methods: proper technique while performing new skills, attention on iliotibial band stretching, flexibility and strengthening of quadriceps and hamstring, and hip abduction and peripelvic strengthening and stabilization [39].

In basketball players, the injury can occur at the end of the match, which is the main moment of fatigue development during the athletic performance. Several authors have reported that the incidence of injuries is higher in the last third of training and games and have hypothesized fatigue is the cause of changes in neuromuscular control [40–42].

Cumps et al. [43] reported the incidence of different causes of anterior knee pain in active individuals: exercise loads being too high (56.7%), monotony of exercise (10%), and previous trauma (3.3%). Furthermore, they found that the player position with highest risk and prevalence was center players (26%). These data confirm what has been demonstrated by Anderson et al. [44] that identified a temporal relationship between training load and injury and reported injuries tend to mostly occur during times of increased training loads.

Basketball is based on specific movements that differentiate its risk factors and mechanisms of injury from other sports. Basketball requires 35–46 jumping and landing activities per game, which is 2–4 times greater than volleyball and soccer [45–48]. More over, basketball players change directions or activities every 2-3 seconds with constant multidirectional acceleration and deceleration [45, 46]. Different than other multidirectional sports, basketball has a greater demand of movements not only on sagittal plane but especially on frontal plane during, for example, defensive action [49].

Therefore, PFPS is not the basketball-related injury that traditionally receives a particular attention. It is known that improving risk factors for a frequent pathology as anterior cruciate ligament (ACL) rupture or ankle sprain might increase the risk for another. For example, the primary goal of most ACL prevention programs is the decrease of hip adduction and internal rotation by strengthening hip extensors and external rotators, but increased external rotation strength may increase the risk of PFPS [50, 51].

According to that, in basketball players, preventive programs are of fundamental importance to reduce the risk of general lower extremity injuries.

The relationship between flatfoot deformity and patellofemoral pain syndrome is a widely held clinical notion/observation that is not strongly supported by research findings; previous investigations have hypothesized flatfoot as a biomechanical variable that could be involved as risk factors for PFPS [52]. Witvrouw et al. found no significant difference in leg-length dissimilarity between the students with PFPS-related pain and those without (*P*=0.355) and foot type (*P*=0.54) [53]. In our study, 23.5% of patients were diagnosed with flatfoot; however, no significant differences were found between the ones with PFPS and the others. These findings, according to previous literature, could suggest that foot and limb alignment should be not considered as an additional risk factor for PFPS. In contrast, other authors have shown how genu valgum, external tibial torsion, and femoral anteversion may be associated with subluxation or patellofemoral malalignment [54]. Considering tibia vara as another intrinsic risk factor for PFPS, we found that many basketball players in our group had abnormal limb alignment [55].

We encountered some limitations in the analysis of our results. First, we performed only a clinical examination without any imaging study (plain radiographs, CT scans, or MRI) [56, 57]. Second, the hours of sport activity were calculated on individually reported data and not according to the duration of training and playing. Third, in our study, the age of the participants ranged from 9 to 17 years, with different moments of the growth process that could have influenced the clinical presentation of PFPS. Last, we did evaluate participants at the beginning of the training season, and no evaluation at the end of the sport season was performed.

## 5. Conclusion

PFPS is a common pathology, and according to commonly accepted theories, muscular imbalance and overuse could exacerbate pain and discomfort in athletes. Our findings show high type and level of sport activity are not related to increase frequency of clinical symptoms related to PFPS.

## Figures and Tables

**Table 1:** Groups' demographics.

Group	Patients	Mean age (years)	Symptoms duration (months)	Side			Sport activity (hours)
				R	L	BIL	
Sample	51	14.3 ± 3.6	1.8 ± 3.6	9	6	36	4.3 ± 1.1
RhythGym group (RG)	21	13.8 ± 3.6	6.4 ± 1.4	3	2	16	4.8 ± 1.1
Basket group (BG)	17	14.2 ± 3.1	5.8 ± 2.1	2	2	13	5.3 ± 1.6
Control group (CG)	13	14.5 ± 4.3	7.1 ± 3.6	4	2	7	2.3 ± 0.8

R, right; L, left; BIL, bilateral.

**Table 2:** Groups' baropodometric analysis.

	Patients	Normal footprint	Cavus foot	Flatfoot	*P* > 0.05
Sample		34	5	12	
RhythGym group (RG)	21	14	2	5	
Basket group (BG)	17	12	2	3	
Control group (CG)	13	8	1	4	

**Table 3:** Groups' clinical knee tests.

Group	Patients	Positive tests	Negative tests	*P* > 0.05
Sample	51	11	40	
RhythGym group (RG)	21	3	18	
Basket group (BG)	17	4	13	
Control group (CG)	13	2	11	

## Data Availability

The data used to support the findings of this study are available from the corresponding author upon request.

## Abbreviations

PFPS: Patellofemoral pain syndrome
RG: Rhythmic gymnastics athletes' group
BG: Basketball athletes' group
CG: Control group
KPS: Kujala patellofemoral score
NF: Normal footprint
FCF: Cavus feet
FFF: Flat feet
MLA: Medial longitudinal arch
PGt: Patellar glide test
PTt: Patellar tilt test
GQ: General quadriceps
VMO: Vastus medialis obliquus
ACL: Anterior cruciate ligament
CT: Computed tomography scan
MRI: Magnetic resonance imaging.
